# Secondary Hemophagocytic Lymphohistiocytosis in a Young Hispanic Adult

**DOI:** 10.7759/cureus.13084

**Published:** 2021-02-02

**Authors:** Bessy S Flores Chang, Carlos E Arias Morales, Marjorie M Flores Chang, Ivette Vigoda

**Affiliations:** 1 Medicine/Nephrology, St. Barnabas Hospital Health System, Bronx, USA; 2 Medicine, City University of New York School of Medicine, New York, USA; 3 Medicine, St. Barnabas Hospital Health System, Bronx, USA; 4 Hematology-Oncology, St. Barnabas Hospital Health System, Bronx, USA

**Keywords:** secondary hlh, t-cell lymphoma, hemophagocytic lymphohistiocytosis (hlh), epstein-barr virus

## Abstract

Hemophagocytic lymphohistiocytosis (HLH) is a disease caused by a severe immune system reaction that involves an overwhelming inflammatory response with overproduction of cytokines and hemophagocytosis. HLH is classified as primary HLH or familial HLH (PHLH or FHLH) and secondary HLH. PHLH is due to mutations in several genes that regulate immune cells, while secondary HLH is triggered by a severe illness (viral infections or malignancies) that induce an excessive immune response that is difficult to control. We present a case of a young Hispanic adult female with a medical history of diabetes mellitus type 1 and hepatitis E that was diagnosed with HLH secondary to lymphoma caused by Epstein Barr virus infection. The patient was started on broad-spectrum antibiotics and steroid therapy; however, the patient succumbed to the disease. HLH is associated with high mortality, mainly because it is not a very common entity and patients usually present critically ill and deteriorate very fast. Immunosuppression and treatment of the underlying disorder is the target of the treatment of HLH, however, the prognosis remains poor.

## Introduction

Hemophagocytic lymphohistiocytosis (HLH) is a rare hematologic disorder characterized by severe immune system dysregulation, leading to the overproduction of inflammatory cytokines, hyperinflammation, and histologic evidence of hemophagocytosis. It frequently affects multiple organs and has been associated with poor prognosis [1]. HLH can be inherited as an autosomal recessive disorder or develop secondary to other pathologies. Although this entity is more common in children and younger adults, it can also present at any age. The incidence rate of HLH is variable, occurring in 1 out of every 3,000 people in North America. It is estimated that about 25% of pediatric cases are primary HLH (PHLH), whereas nearly all adult cases are secondary HLH; the annual incidence rate of PHLH is 1.2 per 1 million children, whereas the incidence of secondary HLH among adults is uncertain [2,3].

Proposed diagnostic criteria include unremitting fever, purpura, hepatosplenomegaly, mental status changes, pancytopenia, coagulopathy, hypofibrinogenemia, lymphadenopathy, liver dysfunction with an elevation of liver enzymes, hypertriglyceridemia, and hyperferritinemia. Laboratory findings may include defective natural killer (NK) cell function and low perforin expression and low erythrocyte sedimentation rate (ESR) [4,5]. In our case, HLH was not initially included in the differential diagnosis, probably due to the lack of awareness of this entity by clinicians. We present a case of secondary HLH in a young adult female patient. The aim of this report is to describe the features of the disease and create awareness of this clinical entity in the general adult population.

## Case presentation

A 38-year-old female from Mexico with a medical history of type 1 diabetes mellitus and Hepatitis E, presented to the emergency department with a one-day history of altered mental status due to metabolic encephalopathy secondary to hypoglycemia that was resolved after administration of intravenous dextrose solution. She reported an eight-month history of intermittent facial edema, periorbital discoloration, fatigue, intermittent fever, diffuse arthralgia, oral ulcers, and unintentional weight loss. She had been undergoing immunosuppressive treatment for presumed dermatomyositis, achieving modest improvement in her facial edema but frequently relapsing after several attempts in tapering immunosuppressive therapy. An extensive rheumatologic workup had been inconclusive. Physical examination was significant for periorbital edema with palpebral violaceous discoloration, hepatosplenomegaly, diffuse adenopathy, arthralgia, and peripheral edema. On admission, the patient was found to be pancytopenic, hypoglycemic, with marked elevated liver enzymes, and lactic acidosis. Broad-spectrum antibiotics and intravenous steroids were started with slight clinical improvement.

**Figure 1:**
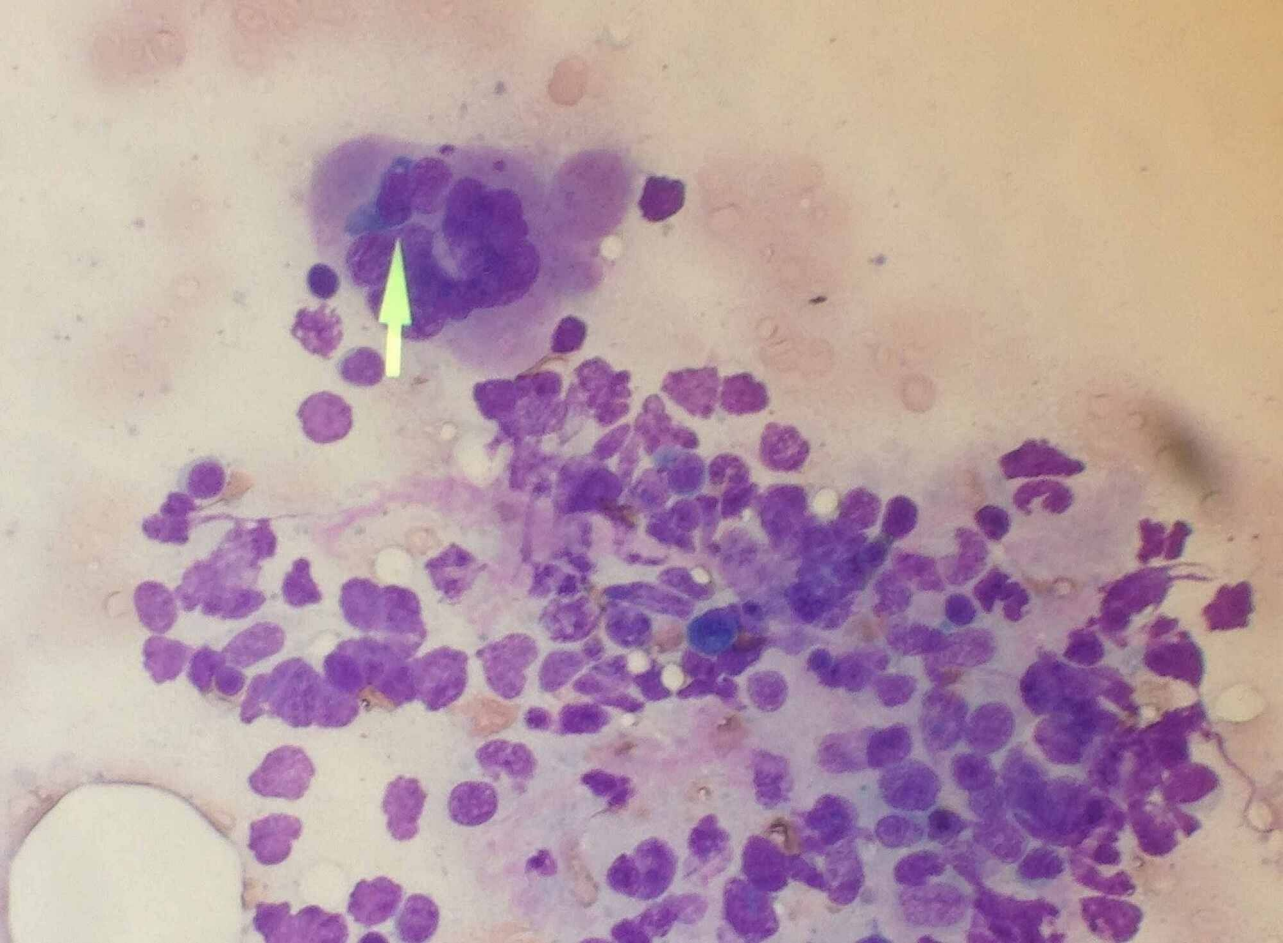
Bone marrow biopsy depicting histiocytic cell in the process of phagocytosis of an erythroid precursor cell.

An extensive infectious and rheumatologic workup was unremarkable and included cerebrospinal fluid analysis, serum, urinary and cerebrospinal fluid cultures, antineutrophil cytoplasmic antibodies (ANCA) vasculitis, rheumatoid factor, anti-smith (SM) and ribonucleoprotein antibodies (SM/RNP), Sjögren antibody, Lyme disease, antinuclear antibody (ANA), double-stranded DNA (dsDNA), and beta-2 microglobulin. A bone marrow biopsy and aspirate were pursued due to worsening pancytopenia with evidence of leukoerythroblastic findings on the peripheral blood smear. Initial findings were remarkable for the presence of macrophages and histiocytic cells hemophagocyting erythroid and myeloid precursors (Figure 1). A spleen biopsy revealed T-cell lymphoma confirmed in the final bone marrow pathologic analysis (Epstein-Barr virus (EBV) positive cytotoxic T-cell lymphoma). Bone marrow fluorescence in-situ hybridization (FISH) analysis showed negative for a t(4:14)/FGFR3-IGH, negative for rearrangement or loss/gain of KMT2A gene. Positive for four copies of 1q21 (CKS1B), negative deletion of DLEU1, DLEU2 genes on chromosome 13 at q14, monosomy 13, and deletion of TP53 on chromosome 17 at p13. Also soluble IL-2 receptor (sIL-2R/sCD25) levels were performed and showed 21,944 U/ml. Unfortunately, after three-weeks since hospitalization, the patient succumbed to the disease.

## Discussion

HLH is a potentially fatal hyperinflammatory condition caused by a highly stimulated but ineffective immune response, that is provoked by excessive activation and proliferation of well-differentiated macrophages, as a result of various different medical conditions, occurring in a heterogeneous group of diseases, ranging from infections or neoplasm to hematological conditions and rheumatic disorders [6].

The pathogenesis of HLH is still not well understood; however, it is thought that the clinical manifestations of the disease are due to hyperactivation of CD8 T-lymphocytes and macrophages; proliferation, ectopic migration, and infiltration of these cells into various organs; and hypercytokinemia with persistently elevated levels of multiple proinflammatory cytokines, resulting in progressive organ dysfunction that may lead to death [7]. PHLH, also called familial HLH, is considered to be a prototype of the hemophagocytic syndrome with a genetic component that results in the inability of cytotoxic T-lymphocytes (CTLs) or natural killer (NK) cells (or both) to efficiently kill target cells [8]. Mutations in the perforin gene are found in 20% to 40% of patients with primary HLH [9]. Secondary HLH designates a macrophage activation syndrome (MAS) that complicates infections, malignancies, or inflammatory diseases such as juvenile idiopathic arthritis (JIA) [10].

According to the International Histiocyte society, five of the following eight criteria are required to make a diagnosis of HLH: fevers, cytopenias of at least two cell lines, evidence of hemophagocytosis, hypertriglyceridemia and/or hypofibrinogenemia, hyperferritinemia, elevated sIL2, decreased NK cell activity, and splenomegaly [10-12]. In our index patient, seven criteria were met.

In a recent study by La Rosée et al., the authors found that viral infections, dominated by EBV, were the most frequent trigger for the development of HLH [13]. In addition, the authors suggest that rapid clinical deterioration is expected in treatment-naive EBV-infected patients, and that treatment with etoposide is mandated without delay. The authors also recommend a more conservative treatment approach with corticosteroids for patients with mild disease or that show an improvement in their clinical manifestations. Furthermore, assessing for treatment response by monitoring ferritin and sIL-2R/sCD25 levels, cell counts, and EBV DNA has also been suggested in the current literature [14].

Immunosuppression and treatment of the underlying disorder is the target of the treatment of HLH. The first international treatment protocol for HLH consists of eight-week induction therapy with etoposide, high-dose dexamethasone, and intrathecal methotrexate for patients with central nervous system involvement followed by maintenance with cyclosporine [4]. Moreover, the addition of rituximab to HLH-directed therapy has been suggested, since it may be effective in clearing the reservoir of virus in patients with HLH caused by EBV. Nevertheless, rituximab should not replace anti-T-cell therapy, corticosteroids, and etoposide [13]. Our patient never received this type of treatment, as she had a fatal outcome during spleen biopsy as part of the work up for the disease.

## Conclusions

HLH is a critical, rapidly progressive disease with high mortality as treatment is often delayed due to the non-specific clinical presentation. Our patient underwent extensive infectious and rheumatologic workup that yielded the final cause of her MAS. Clinicians should consider this entity based on the diagnostic criteria of HLH in patients presenting with febrile illness with pancytopenia, high ferritin, and low fibrinogen levels. Although bone biopsy seals the diagnosis, it should not entertain treatment, especially, if the patient meets the diagnostic criteria. Clinicians also must take into consideration that patients with HLH deteriorate quickly mainly due to potentially fatal complications such as sepsis, multi-organ failure, and bleeding, like in our index patient who developed a hemorrhage after spleen biopsy and rapidly succumbed to the disease. HLH is a treatable entity, however, the overall prognosis remains poor. To date, the optimal treatment for HLH is still unknown but it can vary depending on the severity of the presenting symptoms and the underlying cause. In addition, treatment for HLH remains challenging because the goal of therapies is aimed to decrease the hyperinflammation initiated by the immune system; however, this could also be deleterious for the patient when the triggering cause is related to an infectious agent since the natural defense mechanism to fight the infection could be compromised.
